# Commentary on the Adaptive Significance of Sociality Around Parturition Events, and Conspecific Support of Parturient Females in Some Social Mammals

**DOI:** 10.3390/ani14243601

**Published:** 2024-12-13

**Authors:** Connie Allen Wild, Lisa Yon

**Affiliations:** 1 Centre for Research in Animal Behaviour, College of Life and Environmental Sciences, University of Exeter, Exeter EX4 4QG, UK; 2 School of Veterinary Medicine and Science, University of Nottingham, Sutton Bonington LE12 5RD, UK; lisa.yon@nottingham.ac.uk; 3 Elephant Welfare International, 35-37 Ludgate Hill, London EX4 4QG, UK

**Keywords:** parturition, sociality, social context, social buffering, birth support, labour, health, wildlife, welfare

## Abstract

In recent decades, it has become apparent that during parturition events in a number of social mammals, social support behaviours from group mates can be directed to parturient females (and their newborn neonates). Such behaviour has been documented in diverse taxa, across non-human primates, *Elephantidae*, *Cetacea*, and *Chiroptera*, living in a range of social group organisations, from matrilineal groups to cooperatively breeding groups and multi-male, multi-female groups. Since sociality, in association with parturition, has been demonstrated to confer several health benefits to human mothers and neonates, here, we also consider the potential adaptive significance of social support behaviours for other, non-human, social mammals. If appropriate social environments reduce a parturient female’s dystocia risk and improve her responsiveness to her neonate following a successful birth, then the impacts of the peri-parturient social environment may ultimately have far-reaching impacts on the mother–neonate dyad’s fitness. This seems a logical sequela since the health condition of a neonate at birth and the successful establishment of a strong maternal-neonate bond are often the most critical factors influencing mammalian offspring survival to independence. The principles of kin selection and alliance enhancement may serve to explain the fitness benefits to individuals who support group mates during their parturition and thus the selective advantage conferred to those exhibiting such behaviours. Older, multiparous females appear to hold a particularly important role in the assistance they can provide during the parturition of their group mates, given their greater level of experience of these events. Furthermore, a social birth may have an important influence on horizontal information transfer within a group. In particular, in long-lived, cognitively advanced social mammals (e.g., non-human primates, *Elephantidae*, *Cetacea*), witnessing birth events, early neonate responses, and maternal care, and engaging in allomaternal care with young neonates may be essential for nulliparous females’ normal development. Such events may serve to prepare them for their own parturition and may improve their own parturition-related survivorship and that of their first-born offspring. Thus, it is vital that a better understanding is gained of the importance and salient features of social births in improving the health and survivorship outcomes for both the mother and her offspring in highly social species. The aim of this commentary is to assemble our current understanding of these highly interconnected themes. We suggest in the future, insights gained through observation of non-human social parturition in domestic and non-domestic species, by a wide and highly interdisciplinary range of stakeholders (including zookeepers, wildlife tourism guides, breeders of domestic animals, indigenous people, and ethologists), will be critical for enhancing our understanding of the influence of social environment on this rarely witnessed, yet highly important life event.

## Simple Summary

As an increasing number of birth events have been observed and documented in non-human mammals in recent decades, it has become apparent that for some social mammals, social dynamics during parturition events may be important for reducing maternal anxiety, facilitating labour progression, and improving birth outcomes, maternal-neonate bonding, and aspects of long-term health for parturient females and their impending neonates. The mere presence of group mates may have an important social buffering effect on parturient females, and in some cases, socially supportive behaviours may be performed by group mates toward labouring females and/or their newborn neonates after delivery. In this commentary, after discussing diverse examples of socially supportive behaviours at birth events across a range of mammalian taxa, we also discuss the potential adaptive significance of aspects of sociality around parturition events in some social mammals. We also highlight the importance of further research to enhance knowledge in this field in order to improve both maternal and neonatal health and to contribute to improved long-term population health and fitness in some social mammals.

## 1. Introduction

Anthropological research once popularised the narrative that humans are exceptional in exhibiting conspecific support of parturient mothers and their neonates during parturition events and that non-human mammals isolate themselves to give birth [1,2]. However, over the past three decades, scientific research on the behaviour of captive and wild mammal populations has substantially increased [3]. Furthermore, the increasing popularity of wildlife tourism [4] and a greater spatial overlap between human and wildlife populations [5] have provided greater opportunities to witness rare wild animal life events, such as parturition. In addition, advancements in technology now allow 24/7 remote monitoring of some captive living peri-parturient females, including overnight (many mammals tend to give birth at night or in the early hours of the morning [6]). Increased accessibility to recording equipment and improved means to share digital data [7] have resulted in a steady expansion in the sightings and documentation of the rare and elusive event of eutherian mammalian birth in a wide range of species [3,4,8]. These observations, scattered across the academic and grey literature and social media platforms, have revealed that not only do many social mammals give birth within their social group, but some species also engage in a variety of social behaviours (many of which appear socially supportive) exhibited by non-parturient group mates, directed towards parturient females during labour, neonate delivery, and the immediate postpartum period [9]. Consequently, there has been a shift in perspective, moving away from an assumption of human uniqueness in sociality around parturition events and in expressing supportive behaviours to assist in the parturition of a conspecific [10].

Where non-human parturition support has been discussed, it has primarily been examined in non-human primates [10], often in relation to examining the evolutionary origins of human birth support in the context of other human adaptive features such as bipedalism and encephalisation [10,11]. Furthermore, where non-human birth support is discussed, the primary focus has been on the specific behaviour of a non-parturient physically extracting a neonate from the birth canal of a parturient group mate [10]. However, a broader consideration of the ethology literature suggests evidence for additional behaviours that can be directed to parturient females by their group mates that could also be classified as providing parturition support. These include observations in both non-human primates [12,13] and other taxa, including *Elephantidae* [14,15], *Cetacea* [16,17], and *Chiroptera* [18], as discussed in detail in this text.

Importantly, some otherwise highly social mammals do isolate themselves to give birth, which is thought to be linked to infanticidal threat of group mates or predation threat (e.g., in African lions (*panthera leo*) [19], European wild boar (*Sus scrofa*) [20], and chimpanzees (*Pan troglodytes*) [21]). There are also cases where conspecific presence at birth events appears disruptive rather than supportive; for example, in one study, the majority of witnessed humpback whale (*Megaptera novaeangliae*) births (9 parturitions) involved male harassment of parturient females by groups or individual escorts, which appeared to disrupt important postpartum behaviours such as maternal resting and maternal assistance of neonate breathing and, in some instances, forced mothers to temporarily abandon their vulnerable newborns [4]. Similarly, in one study of ring-tailed lemurs (*Lemur catta*), in 2 out of 9 parturitions, group mate females attacked parturient females after they delivered their neonates [22]. For many social mammals studied, however, parturient females (even those in active labour and those demonstrating behaviours indicative of distress and pain) appeared to make efforts to maintain proximity to their group mates (Mandrills (*Mandrillus sphinx*) [3], Mantled howler monkeys (*Alouatta palliata*) [23], Gelada baboons (*Theropithecus gelada*) [24], Japanese macaques (*Macaca fuscata*) [9,25], and *Bovidae* [26,27], *Pinnipeds* [28,29,30], and *Chiroptera* [18,31]). For many social mammals, giving birth within the safety of the group may be critical in providing protection from predators or harassment from extra-group conspecifics at a time of heightened vulnerability for both the parturient and her imminent neonate [32,33]. Furthermore, in addition to staying in their social group for labour and delivery, some parturient females receive intense interest and diverse focused behaviours from their group mates during parturition events. Indeed, in some cases, rather than the parturient seeking proximity to her group mates, the parturient is mobile and restless, and in such cases, may be actively followed by her group mates who seek to maintain proximity [13,34] and may be defended and protected by group mates when extra-group threats approach her [18,35].

Here, we discuss identified social interactions that appear to indicate that conspecifics are supporting group mate parturient females. These behaviours will be categorised as support given in the prepartum stage (labour and delivery of a neonate) or given in the postpartum stage (time immediately after the birth of a neonate). The prepartum stage may be further divided into “stage 1” of labour (where the cervix dilates and uterine contractions gradually intensify in frequency and magnitude and become more viscerally intense and painful [36,37]) and “stage 2” (where, along with continually intensifying uterine contractions, the foetus is pushed through the birth canal and emerges from the mother’s body [38]). In reality, however, these stages progress naturally from one to the other, with no clear “start” or “end” point [39], and from external observation of animal behaviour alone, distinguishing between these phases can be difficult [9]. Moreover, the earliest stages of labour and initial onset of contractions and maternal discomfort are often not detected by researchers [5,40], particularly for the labours of more experienced, multiparous females [41]. Furthermore, some species give birth by “expulsive delivery” as soon as the foetus has passed the cervix, and there is no clear “stage 2”, or “pushing stage” [15]. The final stage of parturition, occurring after neonate delivery, “Stage 3”, involves placental detachment from the uterine wall and expulsion of the placenta and other remaining foetal membranes from the parturient [42].

There is substantial variation, both between and within species, in the length of total and individual stages of labour [43,44,45], with parity being an important determinant (within species, typically primiparous females have a longer labour than multiparous females [46,47,48]). However, in most mammalian species, there is evidence that shorter labours generally result in improved maternal and neonatal outcomes compared to longer labours [49,50]. Dystocia refers to a prolonged, stalled, or difficult birth owing to either physical blockage (e.g., foeto-pelvic disproportion [51,52,53], including owing to malpresented or abnormally positioned foetuses, the simultaneous presentation of multiples [54,55,56], insufficient cervical dilation [57,58,59], or diseases of the reproductive tract [57,60]), or uterine inertia (e.g., maternal fatigue, overstretched uteri, or compromised myometrium function [47,61,62]). Dystocia can cause foetal asphyxia (interruption to oxygen flow to the foetus), resulting in foetal hypoxia and metabolic acidosis [63,64]. Compared to normal births, those in which dystocia occurs are associated with a greater rate of stillbirth (e.g., owing to asphyxiation in the birth canal) [49,56], neonates with poorer condition and resulting vigour [9,49,56,65,66,67], a greater instance of neonatal complications (such as neonatal infection, birth injuries, meconium aspiration syndrome, and excess postnatal neonatal weight loss [68,69,70,71]), or early neonatal death (including owing to an increased rate of maternal rejection and lactation failures following dystocia births). This increased risk of early neonatal death associated with dystocia births has been seen in a wide range of species, including pigs [64,72], horses [56,73], bats [74], *cetaceans* [34,75], cattle [76,77], sheep [78], *pinnipeds* [79], and primates [80]. Importantly, the directionality of cause and effect between longer labours and some of these negative outcomes can be difficult to discern; for example, intrapartum stillbirth can cause a dystocia, and dystocia can cause intrapartum stillbirth [56,81]. For the parturient, prolonged labours and dystocia are associated with a greater instance of future infertility [82,83,84], maternal peri-natal health complications (e.g., an increased risk of uterine rupture, tearing of the cervix, vulva, vagina, or rectum, infection and inflammation of the reproductive organs, severe haemorrhage, and retained placenta [15,49,85,86,87,88]), and maternal death in affected females [89,90].

## 2. Parturition Within the Social Group

In a number of highly social species, giving birth within close proximity of vigilant and protective group mates may provide the parturient with protection from predators and other extra-group threats when she is otherwise largely incapacitated from such duties herself [12,17,91,92,93]. During labour, some parturient females can be highly mobile and vocal, owing to pain and distress experienced (particularly primiparous females, or those experiencing labour complications [40]), and consequently may be particularly conspicuous to external threats such as predators [32,93,94]. Importantly, even where group mates in the general proximity of parturients may at first observation appear indifferent to the parturition events of their group mates, it is possible that they are in fact highly vigilant, to both external threats and to the condition and behaviour of the parturient, and they may be ready to step in were assistance required. Such social partners may be intentionally giving the parturient the freedom to move as they need, as locomotion and position change are frequently central and critical components of natural parturition in many species [33,95,96,97,98]. In support of this contention, there are various accounts of previously assumed disinterested conspecifics performing aggressive or defensive behaviours in response to a human observer’s approach to a labouring female (Rodrigues fruit bat (*Pteropus rodricensis*) [18] and black-and-white snub-nosed monkeys (*Rhinopithecus bieti*) [35]). Furthermore, in many species, it is common for immature or nulliparous females to pay particular attention to parturition events (e.g. watching the behaviours of the parturient, their neonates, and any supporting conspecifics), which may play an important role in their developing understanding of parturition processes (Japanese macaques [25] and black-and-white snub-nosed monkeys [99]).

In addition to providing actual protection to parturients, the presence of conspecific group mates may also be important in providing the parturient female with a sense of being in a protected and secure environment, hence reducing her anxiety, which is also important in improving parturition outcomes. Whilst rises in maternal cortisol are often observed during normal mammalian parturition [100], exposure to other significant stressors, or fear-inducing stimuli, can disturb, halt, or even reverse labour progression in many species [39,100,101,102,103] through reduced uterine contractility, energy efficiency losses, and slowed cervical dilation [62,101,104]. For example, when experiencing extreme levels of stress owing to disturbances such as sudden noise or the presence of predators during labour, parturient females have been observed to delay labour (blue wildebeest (*Connochaetes tauvinus*) [33]) or retract partially expelled neonates back into the vagina (harbor seals (*Phoca vitulina*) [28]). Epinephrine, which is released from the adrenal gland (along with cortisol) in response to stress or fear, can inhibit the release (and function) of oxytocin—a hormone crucial for stimulating and sustaining uterine contractions during parturition [62,101], or can cause the parturient’s autonomic nervous system to shift to sympathetic nervous system dominance, which stalls labour [105]. Moreover, stressful environmental conditions can stall labour via opiodergic pathways that also inhibit oxytocin release [103,106]. For social mammals, involuntary social isolation at any time can be a significant stressor for individuals [107,108,109,110,111], hence the presence of group mates may have an important social buffering effect, reducing stress in parturient females. In humans, for example, women that go through labour with the continuous presence of a birthing partner report that they feel more confident and less anxious, and evidence shows that they have shorter labours than those that do not have birthing partners [112,113,114,115], or those that are intermittently left alone during their labour [116,117]. Consequently, continuous labour support is listed as one of just 4 key factors for dystocia prevention in humans [50]. We propose, therefore, that for some non-human social mammals too, giving birth in their social groups may reduce parturient anxiety and create an internal hormonal environment more optimal for labour progression. For example, in captive elephants, separation of a parturient from her social group while undergoing labour raises her dystocia risk [118,119,120].

## 3. Potential Social Support Behaviours at Parturition Events

During parturition, social mammals have been seen exhibiting a range of behaviours directed toward parturient females that could be described as parturition support; Demuru and colleagues [13] argued that all the features that define traditional birth attendance in humans are also observed in bonobo (*Pan paniscus*) parturition. With respect to prepartum behaviours, in many species, non-parturients may exhibit affiliative behaviours to parturient females, such as allogrooming, huddling, and other prosocial touching (Japanese macaques [25], capped langurs (*Trachypithecus pileatus*) [93], golden snub-nosed monkeys (*Rhinopithecus roxellana*) [121], black-and-white snub-nosed monkeys [35], titi monkeys (*Callicebus oenanthe*) [96], and Rodrigues fruit bats [18]). Although in many of these studies, rates of exhibition of these behaviours were not compared to baseline rates of their performance to the parturient when she was in a non-parturient state, so we cannot be certain yet whether there is necessarily an increased expression of these behaviours toward individuals in labour, and such comparisons should be an important consideration for future research in this area. However, there are some studies that do report a difference in frequency of expression of these affiliative behaviours compared to baseline rates; for example, the huddling of non-parturients around a parturient female in black-howler monkeys (*Alouatta pigra*) was reported to be atypical of the group’s normal behaviour [5]. Other, potentially important, observations of conspecific social behaviours in the prepartum stage include reports in elephants that parturient females have been observed pushing against group mates for leverage during contractions [122]. Social physical touch is also a critical component of traditional birth support in humans [112,113] and is thought to assist in pain management and anxiety reduction [123]. Similarly, for some non-human social mammals, affiliative tactile behaviours directed to parturient females may ease any anxiety and distress she may be experiencing [124]. Such behaviours may therefore act to reduce the stress response, thus concomitantly reducing the circulation of the associated hormones (which in excess may be detrimental to the progression of labour [125,126]), whilst also potentially promoting the release of oxytocin (which promotes myometrial contractions and labour progression [101,127,128] and modulates the cognitive aspects of how labour pain is experienced by a parturient [129,130]). Myriad studies link physical touch, such as huddling and allogrooming between bonded group mates, with oxytocin and endorphin release, and hormonal profiles indicative of reduced stress in social mammals (general reviews in social mammals [125,126], non-human primates [124,131], and domestic dogs [132]).

Other social prepartum behaviours of potential interest include genital investigations of the parturient by group mates (e.g., Titi monkeys [96], Japanese macaques [25], Rodrigues fruit bats, [18] and Asian elephants (*Elephas maximus*) [133]) and efforts to keep the genital area of the parturient clean (e.g., bonobos [13]). During a fruit bat parturition, Kunz and colleagues [18] postulated that a non-parturient multiparous group mate demonstrated a birthing posture to the parturient; similarly in humans, traditional birth support companions may encourage movement and position changes to a parturient, which can facilitate labour progression and reduce labour pain [123]. Lastly, there are reports of non-parturient group mates gently extracting neonates from the birth canal of parturient females in their group in several non-human primates [35,91,121] and bottlenose dolphins (*Delphinus truncates*) [75]. Similarly, there is preliminary evidence for behaviours that indicate anticipatory neonate-catching behaviours from non-parturients in primates [13] and potentially also in fruit bats [18].

During some social mammal birth events, general group “excitement” increases up to the moment of birth, and in others, there may be an intense escalation in group activity after the actual moment of neonate delivery [17,122,134,135]. For example, in African elephants (*Loxodonta Africana*), as a birth approaches, there can be clustering of herd-mates around the parturient, with intensifying volume and frequency of rumbling and increased ear flapping [122,135]. After calf delivery, there may be a simultaneous release of temporal gland secretions by parturients and non-parturients in the group (temporin release is anecdotally linked to excitement and high-arousal events in elephants [136,137]), continuous rumbling and loud trumpeting during the immediate postpartum period [14,122], simultaneous urination among adult females in the group (C. Allen Wild, personal observation), and tight clustering around the parturient and her calf [14]. Similarly, in various social cetacean births, non-parturient group mates can be highly active in the time immediately after the delivery of a neonate in their group, with reports of fast swimming, thrashing in the water, vigorous leaping, and breaching [17,134,138].

In the immediate postpartum period, vigilance and protection behaviours from group mates may continue to be important for recently parturient females—who may often be occupied with tending to and bonding with their neonates, resting, or consuming the placenta, and therefore less able to be vigilant to external threats [35,121,139]. The newborn neonate too is especially vulnerable to external threats such as predation [140]. In African elephants, group members often gather around the recent parturient and her neonate, adopting defensive postures [14]. There are also reports of non-parturient group mates performing essential afterbirth procedures towards non-offspring neonates, such as severing the umbilical cord [35], breaking intact amniotic sacs [14], encouraging the neonate to stand [14], or, in aquatic species, holding a weak neonate to the water’s surface to assist in breathing [16,17,134,141]. Group mates may also assist the recent parturient in the immediate postpartum period, for example, with food sharing in primates [35], fanning (a cooling behaviour) in fruit bats [18], or caring for the neonate whilst the mother rests [35] or is absent due to retrieving essential replenishing resources such as water [14]. In general, diverse allomaternal care from group mates may be expressed in the immediate postpartum period in some species, and the new neonate may attract substantial interest in the group, with efforts to protect, touch, handle, or lick the new neonate [14,91,92]. At extremes, in elephant species, lactating herd members are even observed to temporarily or permanently adopt and allonurse newborn calves if the neonate’s mother is having difficulties bonding with and caring for her calf [8]. As in prepartum parturition events, nulliparous females are often highly motivated to observe postpartum behaviours of parturients and their neonates [35,99,121]. In primates, group females may try to hold newborn non-offspring neonates, although mothers can be quite selective about whom they allow to hold their neonates, for example, permitting multipara to do so but rejecting the infant handling attempts of nulliparous females [91,92].

Just as the social environment may influence the internal physiological state of parturient females during labour, we suggest that social environments that lower parturient stress and anxiety may also be critical for facilitating the expression of important postpartum behaviours, which affect both maternal and neonate outcomes. Adequately socially supported mothers, with reduced stress, may have better emotional reactivity to their neonates and an improved capacity to bond with and care for them [142,143,144,145,146,147,148,149] compared to females with inadequate postpartum social environments. In some social species, appropriate social environments can buffer stress-reactive aggression in vulnerable individuals [150,151,152]. Along with the hormonal surges and fluctuations at the time of parturition, the general stress and pain that a parturient female experiences can influence their mental state, and some social mammals, particularly those that are prone to stress-reactive aggression to novel stimuli, can exhibit maternal aggression to neonates that can be lethal, particularly during primiparous parturition (e.g., elephants and pigs [8,118,153,154]). In these species, it has been suggested that primiparous parturients with more appropriate social environments, with greater exposure to multiparous females, and greater prior exposure to birth events in their groups, will have less neophobia of neonates, reduced stress at parturition, and exhibit a reduced risk of maternal aggression to their neonates after birth [8,118,120,153,154]. Additionally, experienced multiparous females present in the group may be able to actively intervene to manage any aggression of parturients to their neonates. In elephant family groups, immediately after a parturition, the matriarch may push aside parturients to watch over non-offspring newborn calves themselves [155] or direct prosocial contact to parturients, which appears to ease maternal distress and subdue maternal aggression to neonatal calves (C. Allen Wild, personal observation). This is supported by other elephant research that reports that multiparous group mates can assist primiparous mothers in learning to accept initially rejected calves [8,156]. In contrast to this, in some species, there are reports of non-parturient group mates exhibiting aggressive behaviours to newborn neonates after birth, for example, flipping the neonate out of the water in killer whales (*Orcinus orca*) [134] and aggression from conspecifics to newborn calves in bottlenose dolphins [138]. However, such behaviours are performed within the context of what seems to be a state of high arousal and excitement in non-parturient group mates postpartum, and although these aggressive behaviours have the potential to be injurious, both calves survived in the cited examples.

Of the discussed examples of potential pre- and postpartum social birth support, there appear to be consistent patterns regarding who receives and who performs particular birth support behaviours. For example, nulliparous subadult and juvenile females that often watch birth events at close range may provide a beneficial social buffering effect to the parturient, despite not interacting directly with her, and any attempts of these immatures to interact with newborn neonates directly after birth will often be blocked by post-parturient mothers [35,99,121,157]. Similarly, in groups with males present, males typically only engage in vigilance and defensive behaviours, which serve to protect the parturient from external threats (e.g., threatening extra-group threats that approach parturient females [18,35,158]), although in primate groups where males have a high degree of certainty about their paternity, males are observed allogrooming the parturient during labour [35,96]. However, for most of the social birth support behaviours discussed that involve direct interaction with the parturient, such as intimate prosocial touching of the parturient [12,91,92], assisting with neonatal extraction [35,91,99], severing of the umbilical cord [35], rupturing and removing foetal membranes [14], and providing allomaternal care to neonates postpartum [91,92], primarily multiparous females have been observed performing these interactions, which may require specialist knowledge. Such behaviours may be linked to the fact that older, multiparous females have more experience in the birth process. For many long-lived social mammals, older individuals hold important roles in their groups due to their accumulated knowledge, which they can communicate to the benefit of their group mates [159,160,161,162]. For some social mammals, the ability to provide effective birth support to group mates may be a skill developed across a female’s life as she experiences more parturitions herself and that of others in her group [24]. In addition to providing practical assistance in the birth processes of their group mates, older multiparous females may also be particularly effective social buffers for parturient females [150,151,163]. In other words, parturients that give birth in the company of experienced multipara may have a reduced level of anxiety (and the associated, previously discussed, benefits of shorter and less complicated labours and better reactivity and bonding with their neonate, leading to improved short- and long-term maternal and neonatal outcomes as a result) as they perceive that they are in a safe environment, in the company of individuals that have specialist knowledge of how to skilfully assess risks to determine how, when, and if to intervene to assist the parturient and her neonate(s) [162]. In traditional human societies too, the role of birth attendance is typically held by older, multiparous women [164,165,166]. Of similar note, in most of the above-cited examples, it is primiparous females who are receiving the reported birth assistance, potentially reflecting the observation that primipara are often more overtly distressed, restless, and panicked during labour than multipara [40,41,97,167] and are more likely to have difficult labours [24,47,48,168]. Nevertheless, there are also reports of multiparous females receiving supportive and affiliative interactions from group mates during labour [5].

## 4. Adaptive Explanations for Social Support Behaviours at Parturition

The presence of birth support behaviours across various mammalian taxa suggests it holds evolutionary significance, and sociality around birth events may confer several adaptive advantages to parturient females, their neonates, and the individuals present at births or interacting with the parturient. For parturient females and their neonates, compared to giving birth alone, females that give birth in their social groups may directly benefit from the vigilance and protection behaviours of group mates, improving their own and their neonate’s survival probability in a time of heightened vulnerability to predators and extra-group threat [32,94,169]. Linked to this, as mentioned, labouring females may perceive themselves to be in a safe and well-protected environment when in the presence of group mates, particularly multipara, who have more experience in birth events [25,157,170,171]. This, along with the performance of any comforting behaviours such as allogrooming and other prosocial behaviours by group mates towards parturients, may reduce maternal anxiety and facilitate oxytocin release, which promotes myometrial contractions [113], increases maternal pain thresholds [129,130], and regulates the concentrations of hormones related to stress responses in the parturient’s body that may hinder labour progression [62,104,125]. As a result, appropriately socially supported parturients may have a smoother progression of labour with a lower risk of adverse birth outcomes, such as dystocia and intrapartum stillbirth, maternal death, or poor initial neonatal vigour/condition [3,56,64,73,77,120]. Such initial poor neonatal condition at birth can lead to cascading negative effects on early development such as lactation difficulties (i.e. stressful labour, particularly cases like dystocia, can render newborns with low vigour, making it challenging for them to effectively suckle after parturition; this can compromise the whole lactation process, given that well-established lactogenesis relies on the feedback within the mother–neonate unit and adequate emptying of mammary glands [9,71,72,172,173]), increased risk of hypothermia and starvation, poor neonatal growth, and failure of passive transfer of immunity, thus resulting in greater rates of early neonatal death [67,77,174,175] and reduced chance of reaching weaning age [176]. In humans, for example, women that give birth with the continuous support of a non-clinician companion during labour have shorter labours [114,115,177,178], are less likely to require an emergency caesarean section [178,179,180], give birth to neonates with higher Apgar scores [180], have infants that are less likely to require a stay in a neonatal intensive care unit [177], and are less likely to have maternal mental and physical health complications postpartum [117] than those who labour without such social support.

In addition, as discussed above, females that give birth in optimal social environments may have a better initial response to and bonding with their neonate(s) [146,148,149]. This leads to improved overall fitness for the parturient, owing to improved offspring survival—with the strength of the mother–neonate bond and quality of maternal care received by neonates being the most critical factors determining offspring survival to independence in most mammals considered [120,181,182,183,184,185]. Furthermore, for some social mammals, greater social support in the early postpartum period may improve neonatal survival through improved lactation success (e.g., increased likelihood of the mother–neonate dyad successfully establishing lactation, greater maternal milk yields, higher maternal milk quality, and improved ability for the neonate to achieve appropriate feeding bout frequency and durations [172,186,187]). Lactation benefits afforded to social mammal females (and their dependent neonates) with strong social ties are thought to result from both (i) perceived support and a feeling of safety and security, which reduces maternal anxiety and stress (which can improve milk letdown and milk yields [172,188,189,190]), in addition to (ii) the actual benefits of associating with social partners, especially those with more experience. The latter includes, for example, an improved foraging efficiency and utilisation of environmental resources (which can improve milk quality and yields), social knowledge gains such as the transfer of knowledge of appropriate lactation behaviours, access to temporary or permanent allolactation support from group mates, and provision of general allomaternal support that can relieve maternal stress and allow activity budget changes that enable lactating mothers to dedicate more resources to milk production [8,115,191,192,193,194,195,196]. For example, in elephants, the observation that calves born in families with more allomothers have higher calf survivorship is thought to be owing to an improved foraging efficiency (and subsequent improved milk production) in mothers that have other females supporting with maternal care duties such as vigilance, socialisation, and defence of calves [197,198]. These benefits are particularly helpful for primiparous females, who are often also younger and still growing, hence have less energy to allocate to milk production [198,199]. Similarly, again in humans, women that have social support companions during parturition and postpartum have better bonding with, and responsiveness to, their neonates [117,200], lower rates of postpartum depression and anxiety [180,201,202] (with impaired mother–neonate bonding also more likely to be observed in mothers with postpartum depression [203,204,205]), and improved breastfeeding success [115,191,206,207].

In cases where extraction assistance is needed owing to stuck, atypically presenting, or malpositioned foetuses, the immediate survival of the neonate and/or mother may be secured by the presence of experienced group mates that can manoeuvre and remove neonates gently from the birth canal [35,75,91,92,99,208].

In some species, females attending births are observed to keep the birth environment, the mother’s genital area, and the neonate clean [13]. These behaviours may reduce both the mother and neonate’s risk of infection and death, a significant risk for many species in the peri-parturient and postpartum period [209,210,211]. Alternatively, group mates may be monitoring chemical information in genital secretions or visually assessing the genital region (e.g., for the emergence of a neonate), which may provide information on labour progression and may signal to group mates to perform appropriate, timely social support or interventions [18,25,96,133].

Finally, giving birth within the social group may facilitate a neonate’s social integration into their wider group and its establishment of bonds with potential allomothers, which may improve their survivorship probability and the overall fitness of parturients and neonates. Being socially well connected and integrated in a group confers health, welfare, survival, and overall fitness advantages to many social mammals (e.g., elephants [212] and non-human primates [213,214,215,216]; review on adaptive value of sociality to mammals in general [217]), especially to neonates in species that exhibit a high degree of allomaternal care (e.g., elephants [197,218] and meerkats (*Suricata suricatta*) [219,220]. For example, as previously mentioned, in elephant species, calves in groups that have more allomothers have an improved survivorship [197,198], and the presence of a calf’s maternal grandmother in the group has a particularly positive influence on the survivorship of calves born to primiparous mothers [199,218]. Additionally, for parturients that reject or are separated from their neonates, have injuries that compromise their ability to perform maternal care, or are having difficulties with producing or expressing colostrum/milk after birth; neonatal survival chances are substantially increased in the case where allomothers in groups temporarily or permanently adopt non-offspring neonates [8,221,222,223,224,225,226]. Many social species exhibit an attraction to neonates [35,41,227], and whilst in some species, mothers can be particularly protective of their newborns and may even be aggressive towards group members showing interest in their young, mothers in more socially tolerant species often allow group mates to have close or direct contact with their neonates [14,41,92,228], or at least those that have experience in neonate care themselves [91,93,121,229]. For mothers, oxytocin is understood to be a critical hormone involved in mother–neonate bonding after birth [230,231,232], but it is also possible that the establishment of the neonate into an extended network of allomothers and caretakers may also be mediated by physical contact with, and oxytocin (or other neuroendocrine regulators, e.g., prolactin and vasopressin [233]) release in, non-mothers in the group after birth [32,96,234,235,236]. As such, the immediate postpartum period (which, as discussed, can be an extremely high-arousal event for many social mammals [14,17,122,134]) may be a particularly effective and sensitive time window for optimal social integration of neonates in groups that have a high degree of allomaternal care [236]. For example, in humans, fathers that engage in skin-to-skin contact with their babies in the first days postpartum have higher oxytocin levels, are more attached and responsive to their infants, and are more involved in infant care than those that do not engage in early skin-to-skin contact [237,238,239,240].

There are also likely adaptive benefits to the conspecifics that provide the birth support behaviours discussed. Kin selection is likely to explain many instances where females provide birth support in socially tolerant, matrilineal social groups with close bonds between related females [92,99,241]. For example, in many of the births discussed, it is mentioned that key attending females are known to be closely related to the parturient (sisters, daughters, aunts, cousins, or mothers) [25,35]. Similarly, males providing birth support behaviours discussed are often living in pair-bonded or one-male groups where their paternity of the neonate is more certain [35,96]. In these circumstances, providing parturition support may improve the parturient female and her neonate’s health and survival probability in the vulnerable perinatal period through many of the benefits (to mothers and neonates) mentioned above and hence provide inclusive fitness benefits to relatives who are tending to parturients during parturition events [242]. Importantly, however, birth support behaviours are also observed in non-kin-bonded female groups, such as bonobos [12,13] and fruit bats [18]. Bonobo females have strong, long-lasting, affiliative relationships with non-related females in their groups [243,244], and it has been suggested that heavy investment in maintaining social relationships and reciprocity for future social services may underlie birth support in this species [12,13].

Presence at birth events provides an important benefit through the potential knowledge and experience that can be gained, especially for young nulliparous females who have not yet given birth themselves. Rather than being solely instinctual, many maternal skills in more long-lived, cognitively advanced social mammals appear to largely be learnt behaviours that require practice, and can be transmitted or enhanced through social learning processes between individuals in groups of overlapping generations [161,162,245,246,247,248]. In primate births, juvenile and subadult females often follow females in labour and are particularly interested in watching parturition events (e.g., gelada baboons [249], gray langurs (*Presbytis entellus*) [250], patas monkeys (*Erythrocebus patas*) [251], Japanese macaques [25], and snub-nosed monkeys [99]). In many species, both wild-living and under human care, birth-related mortality (of both mothers and neonates) and failure of the mother–neonate bond (a key determinant of early neonatal death) are significantly greater for primiparous females compared to multiparous females [252,253,254,255]. This higher mortality is often attributed to the inexperience of first-time mothers and the greater hormonal, psychological, physiological, morphological, and behavioural changes that occur in the short peri-parturitional period for females experiencing their first births, compared to multipara [46,78,97,256,257]. For example, in some species, multiparous mothers spend more time in postures that facilitate labour progression [40] and are less likely to have labour complications [258], including dystocia [47,168,259], compared to primipara. After birth, multipara also respond more appropriately to initially irresponsive neonates [9,23], are more skilled at manoeuvring neonates and encouraging lactation [40,41,248,260], are less likely to reject their neonates [33,120,195,228,255,261,262], are more skilled at protecting and accessing the abilities of neonates (so as to avoid accident-related neonatal mortality) [199,263], and have greater overall lactation success [264,265,266,267] compared to primipara. However, “experience” of parturition is not only influenced by an individual’s parity alone, but also the parturient’s prior exposure to birth events and neonate care in her group when she was younger, as well as her own experiences of being mothered. To contrast, animals raised in social isolation or that experience early social deprivation or maternal loss often have problems raising their neonates—for example, poor success in establishing lactation or an inability to bond with their neonates [184,268,269,270]. Birth is a rare event; by observing parturition in their social group and actively participating in allomaternal behaviours during the early postpartum period of group mates, nulliparous females may acquire knowledge that can better prepare them for their own future first experience of labour and delivery. This may include knowledge of how to appropriately respond to neonates after birth (e.g., fear of novelty is thought to underlie various aspects of poor maternal care and poor bonding success in primiparous females with insufficient exposure to neonates prior to their first births [8,40,120,153,154]), as well as providing practice in/exposure to skills such as how to manoeuvre and care for neonates and how to encourage lactation [9,40,41,184,195,233,267,271,272]. For example, primiparous vervet monkeys with prior experience handling infants in their nulliparous years have greater first-born survivorship than those that do not have such experience [245], and captive primiparous elephants with no experience of birth or young calves in their groups are more likely to reject their calves than those with such experiences [120]. It has even been proposed that in long-lived, highly social species, any neophobia shown by primiparous females to neonates at birth may reflect abnormal social development and an unnatural lack of prior access to experiences of birth events and to neonates in their social group [273]. Furthermore, females that have previously witnessed birth events at close range in their groups may be more informed at their own first births and thus have lower anxiety and consequently experience smoother labour progression and improved birth outcomes, as discussed above.

It is not always clear what the advantages are for individuals attending the birth of another, and providing support behaviours to parturient group mates. Placentophagia is observed in nearly all terrestrial mammals [274], and despite a few instances of mothers sharing the placenta with attending females, this food-sharing behaviour is not argued to be a key adaptive advantage for those providing birth support behaviours [12,35,99]. Further hypotheses for the adaptive benefits to conspecifics include that tending to parturients in a prosocial manner (e.g., with allogrooming and proximity) may simply represent a general consolatory response of social mammals to conspicuous signs of distress in group mates [93]. Similar to “soothing” allomothering behaviours directed to some young animals [197,275], group mates may simply be trying to consolidate mothers so as to reduce the conspicuousness of the group to external threats and improve the general group cohesion [276]. Furthermore, for many social mammals, observation of distress in bonded group mates can trigger a stress response in other members of the group [277,278,279]; therefore, there may be motivation to alleviate distress in parturient group mates to reduce the stress of conspecific “bystanders” of the parturition.

## 5. Conclusions

As our understanding of the diverse and far-reaching impacts of the social environment on parturition in social mammals has improved, it has become apparent that there are important implications for the health, welfare, and reproductive success of breeding females (and their offspring) managed under human care. Historically, many captive social mammals were routinely isolated from their groups for their parturitions [40,118,280,281]. However, in recent years, captive animals are increasingly being managed in ways that are informed by the behavioural ecology of that species in the wild [282,283], and in many cases, efforts are now made to allow females to birth naturally among their social groups, where deemed appropriate for the species and the individual parturient [8,284,285]. In the coming decades, these social births in captive animals will supplement our understanding of the natural behaviours of social mammals during a parturition event in the social group, and allow for a more qualitative and quantitative assessment of the behaviours and a more experimental approach to exploring the notions presented in this commentary. The remote monitoring (e.g., with 24 h video surveillance) of parturient females that is possible in captive environments can provide an insight into a rare event that is nearly impossible to see in the wild (particularly so for births that happen in dark hours [3,40], or in environments with poor visibility of subjects owing to habitat features [17]). Captive populations also allow for easy access to baseline samples of the behaviours and activity budgets of parturient females and their non-parturient group mates, enabling critical comparisons of social behaviours between group members in a non-parturient state and a parturient state (e.g., are comforting prosocial behaviours directed to parturient females actually different from her baseline receipt of these behaviours in a non-parturient state?). Furthermore, the continuous monitoring of captive animals allows for retrospective assessment of the behaviours of parturients and their group mates, this is beneficial since, as mentioned, often the earliest signs of labour go initially undetected by human observers in wild studies [5,14]. Finally, many animals under human care have regular hormonal assessment during pregnancy, making parturition timing more readily predictable and observable [286].

Anecdotes from individuals who have witnessed social mammal births in captivity can be highly valuable [75], and in the future, efforts should also be made to collect information from diverse groups of individuals who hold accounts of non-human parturition in captive and wild contexts, including knowledge possessed outside of academic spheres, such as among zookeepers, wildlife tourism guides, breeders of domestic animals, and indigenous people [4,287,288]. Studies suggest that for many more rare behaviours and events in nature, there is a bias against reporting in academic journals, and methods such as surveys of experts can be important tools for collecting data that are otherwise only currently held as tacit knowledge among individuals or documented across the grey literature and other diverse digital media [7,289,290,291].

## Data Availability

Not applicable.
